# Impact of Virtual Reality-Based Therapies on Cognition and Mental Health of Stroke Patients: Systematic Review and Meta-analysis

**DOI:** 10.2196/31007

**Published:** 2021-11-17

**Authors:** Qi Zhang, Yu Fu, Yanhui Lu, Yating Zhang, Qifang Huang, Yajie Yang, Ke Zhang, Mingzi Li

**Affiliations:** 1 School of Nursing Peking University Beijing China; 2 Department of Computer Science Aberystwyth University Wales United Kingdom; 3 Department of Psychology Shanxi Datong University Datong City China

**Keywords:** virtual reality, stroke, cognition, depression, mental health

## Abstract

**Background:**

Stroke remains one of the major chronic illnesses worldwide that health care organizations will need to address for the next several decades. Individuals poststroke are subject to levels of cognitive impairment and mental health problems. Virtual reality (VR)-based therapies are new technologies used for cognitive rehabilitation and the management of psychological outcomes.

**Objective:**

This study performed a meta-analysis to evaluate the effects of VR-based therapies on cognitive function and mental health in patients with stroke.

**Methods:**

A comprehensive database search was performed using PubMed, MEDLINE (Ovid), Embase, Cochrane Library, and APA PsycINFO databases for randomized controlled trials (RCTs) that studied the effects of VR on patients with stroke. We included trials published up to April 15, 2021, that fulfilled our inclusion and exclusion criteria. The literature was screened, data were extracted, and the methodological quality of the included trials was assessed. Meta-analysis was performed using RevMan 5.3 software.

**Results:**

A total of 894 patients from 23 RCTs were included in our meta-analysis. Compared to traditional rehabilitation therapies, the executive function (standard mean difference [SMD]=0.88, 95% confidence interval [CI]=0.06-1.70, *P*=.03), memory (SMD=1.44, 95% CI=0.21-2.68, *P*=.02), and visuospatial function (SMD=0.78, 95% CI=0.23-1.33, *P*=.006) significantly improved among patients after VR intervention. However, there were no significant differences observed in global cognitive function, attention, verbal fluency, depression, and the quality of life (QoL).

**Conclusions:**

The findings of our meta-analysis showed that VR-based therapies are efficacious in improving executive function, memory, and visuospatial function in patients with stroke. For global cognitive function, attention, verbal fluency, depression, and the QoL, further research is required.

**Trial Registration:**

PROSPERO International Prospective Register of Systematic Reviews CRD42021252788; https://www.crd.york.ac.uk/prospero/display_record.php?RecordID=252788

## Introduction

Stroke is the second-highest cause of death worldwide and a leading cause of disability, contributing to approximately 3 million cases every year in China [1]. Survival rates of stroke have improved steadily over the past 2 decades; however, a longer survival length is often accompanied by the sequelae of long-term effects [2,3]. One such long-term effect of stroke is cognitive impairment [4]. It is estimated that approximately 80% of stroke survivors may experience new onset or worsening of cognitive impairment during their recovery [2,3]. Brain changes can affect 1 or more cognitive domains, including spatial awareness, praxis, perception, memory, language, and executive function [5]. Stroke-related cognitive deficits may interfere with functional recovery, the ability to (re-)acquire motor skills, and compromise independence [4,6,7], potentially exerting considerable influence on rehabilitation outcomes. Although stroke occurs as an acute event, it is a chronic condition that necessitates multidimensional and overwhelming treatment [8]. The neurological trauma of stroke survivors is irreversible and devastating; hence, after a stroke, patients face various stressors, which may trigger different aspects of their health, especially their mental health [9]. Mental health is defined as a state of complete happiness, which refers to our ability to enjoy life and cope with challenges [10]. Depression, anxiety, and stress are particularly common and persistent psychological problems following a stroke, with a high risk of relapse, even after a long period of remission [11]. Approximately 22%-40% of stroke survivors are affected by depression symptoms, 9.4%-36.7% by an anxiety disorder, and around 31% by poststroke stress [12]. Multiple studies have also shown that due to unsatisfactory sequelae and irreversible prognosis, patients with stroke always experience higher levels of emotional distress and ineffective coping than the general population, which poses adverse effects on the rehabilitation process and indirectly affects the patients’ quality of life (QoL) [13,14].

Given the cognitive and psychological impacts on stroke survivors, strategies to support these individuals are considered a priority. Targeted rehabilitation can help address the clinically important cognitive and psychological consequences of stroke. However, the effect sizes of conventional rehabilitation are moderate at best, with high dropout rates, as patients, especially those with cognitive and psychological impairments, use rehabilitation services less efficiently and show less adherence to required changes in their lifestyle [15,16]. With underscored inadequacies of conventional rehabilitation, new high-tech innovations that use virtual reality (VR) are considered a potential avenue toward effective rehabilitation and may offer a supplementary platform to enhance the cognitive and psychological benefits after stroke.

VR is defined as a user–computer interface created with computer hardware and software, enabling the user to simulate interactions with environments that appear and feel similar to real-world objects and events through multiple sensory canals [17]. The term “virtual reality” was first coined in the late 1980s [18]. Until the late 1990s [19], VR-based rehabilitation was more widely used and sophisticated within health care systems as VR technology became more readily available and affordable [20,21]. Multiple recent studies have shown the positive role of VR in several clinical conditions, including acute pain [22], social anxiety disorder [23], and chronic obstructive pulmonary disease [21]. With the characteristics of immersion, interaction, and imagination, the application of VR in neurorehabilitation has also been growing rapidly in the recent years [24,25]. Currently, the neurorehabilitation applications of VR have been introduced in the field of neurorehabilitation for Parkinson’s disease, Alzheimer’s disease, brain injury, cerebral palsy, unilateral spatial neglect, and, especially, stroke [26-28]. Management of VR-based neurorehabilitation for stroke is highly related to recovery, reorganization, and neuroplasticity. VR can also exploit a brain mechanism known as embodied simulation, encouraging the patients’ motivation and participation by allowing physical and emotional interactions with the environment through the digital medium [29]. In particular, VR offers a high level of flexibility and control over therapeutic tasks by automatically recording and tracking the user’s performance [30,31], enabling the user to perform intensive training according to their ability and keeping the experience of interaction with therapeutic tasks enjoyable and compelling [25,32].

Due to tremendous benefits for health and the relatively inexpensive medium for rehabilitation training, a significant amount of work focusing on the effects of VR has been proposed for the rehabilitation of deficits following stroke [8,25]. Multiple recent randomized controlled trials (RCTs) on the effects of VR have supported the use of VR in stroke rehabilitation to improve cognitive and psychological outcomes [33-36]. In contrast to studies supporting VR-based rehabilitation, some researchers have argued that there are no or only minor effects detected in global cognitive function, memory, depression, or the QoL [34,37,38].

Although there are several systematic reviews conducted on the contribution of VR-based interventions to cognition rehabilitation, no unified conclusion has yet been reached. Anna et al [25] conducted a meta-analysis by summarizing RCTs published before June 2017 and supported VR as an adjunct to cognition rehabilitation. However, the number of included studies was only 2, suggesting a tenuous finding.

Another recent review [39] extensively searched relevant trials conducted before November 2019. It examined the effectiveness of VR-based therapies on both global cognition and domain-specific cognition poststroke, suggesting that VR therapy is not superior to control interventions in improving both global cognition and domain-specific cognition in patients with stroke. However, their conclusions were also merely based on data from 2 studies, resulting in insufficient statistical power. A systematic review by Zhang et al [40], only focusing on global cognitive function, also supported no benefits of VR for cognition. Furthermore, the psychological benefits of VR have also received more attention recently. A systematic review [8] indicated that exercise-based VR interventions are potentially valuable as a support in improving psychological outcomes. Nonetheless, due to the diversity of study design and insufficient data, the authors only described their results and did not conduct a meta-analysis. Hence, the overall effect of VR-based therapies on improving cognition and mental health in patients with stroke remains unclear.

The evidence is sparse, and a comprehensive picture of the effects of VR-based therapies is needed. With ongoing advancements in VR, a plethora of original studies focused on cognitive recovery and mental health after stroke has been recently published. This makes it possible to review VR applications and guide future design and implementation of VR technology in clinical practice. Thus, our review aimed to comprehensively examine the effects of VR-based therapies on cognition, the QoL, and depressive symptoms in patients with stroke.

## Methods

This systematic review was registered with Prospero, the International Prospective Registry of Systematic Reviews (registration no. CRD42021252788). We conducted this systematic review according to the Preferred Reporting Items for Systematic Reviews and Meta-Analysis (PRISMA) statement.

### Literature Search Strategy

RCTs from PubMed, Embase, MEDLINE (Ovid), Cochrane Library, and APA PsycINFO databases were comprehensively searched. RCTs published in English before April 15, 2021, investigating the impact of VR-based therapies on the cognition and mental health of patients with stroke were included. In addition, we conducted reference tracking on the published trials and meta-analysis reviews in this field to make sure all relevant studies were included. The search strategies, including terms for stroke, VR, cognition function, the QoL, and depression, are presented in Multimedia Appendix 1.

### Inclusion Criteria

#### Types of Trials

We only included RCTs that were peer reviewed and written in English. Reviews, single-case studies, dissertations, conference papers, and abstracts were excluded.

#### Types of Participants

Patients aged 18 years or older with stroke were included. Patients with stroke were identified by any available diagnostic criteria, such as brain computed tomography, magnetic resonance imaging, or other standards and consensus by clinicians. To maximize the number of meta-analyses, we did not restrict the search to any specific stroke population.

#### Types of Interventions

VR-based interventions include either single-component interventions or multiple-component interventions for patients with stroke. VR had to consist of a screen or a head-mounted device, including games with immersive, semi-immersive, and non-immersive systems, simulating virtual environments using computers, video consoles, mobile apps, and VR. The intervention setting, duration, and frequency were not restricted. Participants in the control group could undergo usual care or non-VR interventions.

#### Types of Outcome Measures

The primary outcomes were global cognition and domain-specific cognition (eg, attention, executive function, memory, psychomotor speed, verbal fluency). According to Isabelle Dor’s [10] classification, the components of mental health include emotional well-being/QoL and psychological and social well-being. Our systematic review specifically included depression, stress/distress, anxiety, coping competence, overall mental health, the QoL, and self-efficacy.

### Study Selection and Data Extraction

Two reviewers independently assessed studies obtained from the database searches in three phases: title, abstract, and full-paper screening. Data were extracted by the same two reviewers from trials using a Microsoft Excel spreadsheet. The information extracted from each trial included the first author, year, setting, sample size, participant ages, details of intervention and the control, outcomes, and corresponding measures. Disagreements were resolved through a discussion with a senior investigator.

### Quality Appraisal

The Cochrane risk-of-bias tool was used to assess the quality of eligible trials, focusing on sequence generation, allocation concealment, blinding, attrition bias, completeness of outcome data, and other sources of bias. Any disagreements were settled by a discussion with a third person.

### Data Synthesis and Analysis

The standard mean difference (SMD) and weighted mean difference (WMD) with a 95% confidence interval (CI) were determined for continuity data. Statistical heterogeneity was assessed using the Cochran Q test and the Higgins *I^2^* statistical test. The results implied low-level heterogeneity when *I^2^* ＜ 50%, and we used a fixed-effects model to fix the issue. The results implied moderate or high heterogeneity when *I^2^* ≥ 50%, and subgroups were determined based on the different characteristics of the studies to identify the sources of heterogeneity. Sensitivity analysis was performed by comparing the effect sizes and CIs of the remaining RCTs after removing each included RCT at a time. Descriptive analysis was used to deal with nonmergeable data. Potential publication bias was assessed with funnel plots and the Egger test if the group included 10 trials or more [41]. All meta-analyses were performed in RevMan 5.3 (Nordic Cochrane Center, Cochrane Collaboration, Copenhagen, Denmark) for outcomes that were evaluated in at least 2 of the included RCTs.

## Results

### Study Selection

**Figure 1:**
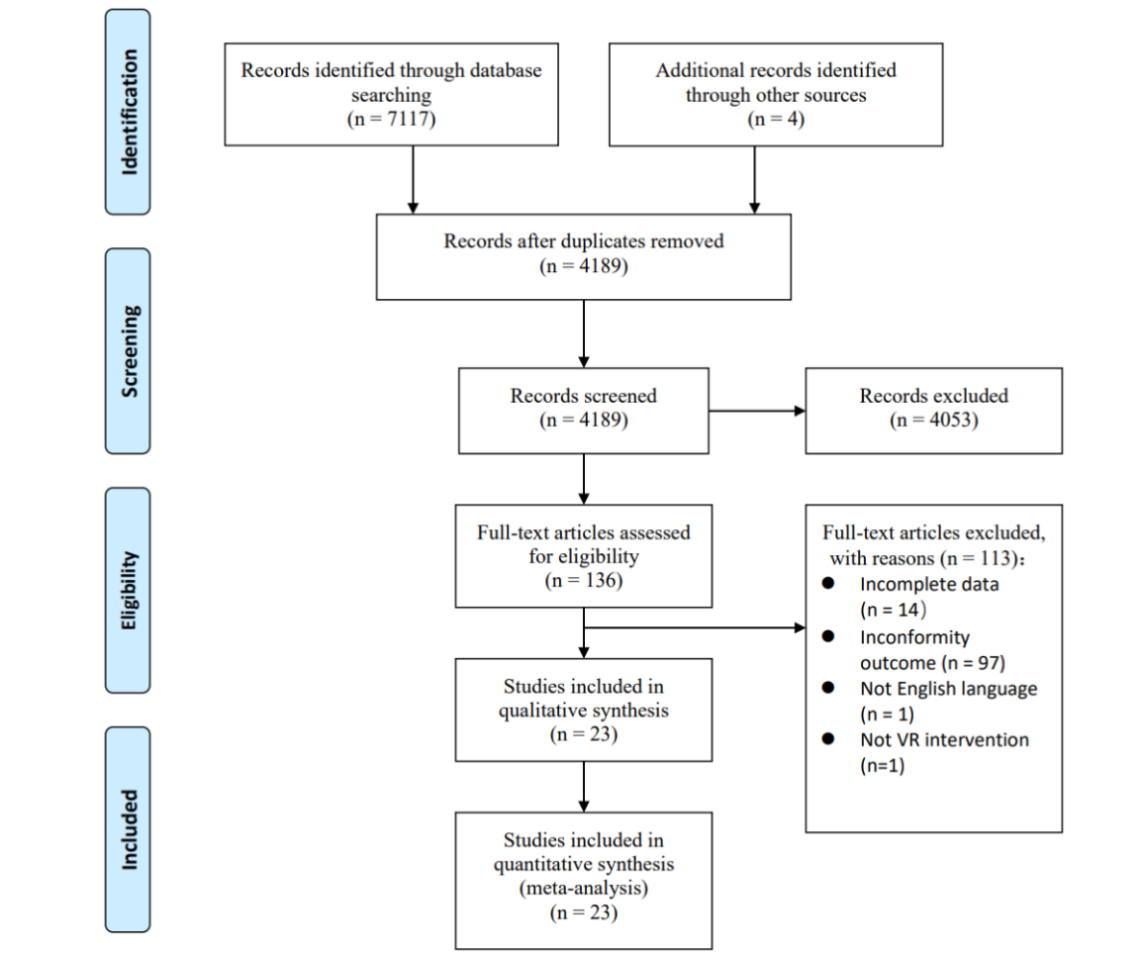
Flowchart of the study selection process. VR: virtual reality.

A total of 7117 studies were identified from the 5 databases searched. A further 2932 studies were identified through manual searching, including searching for systematic reviews, and included studies’ reference lists. After 261 duplicates were removed, 4189 full-text manuscripts were identified by screening their titles and abstracts. After full-text reviews, 23 studies (including 894 participants) from 8 countries satisfied the inclusion criteria and were eventually included in our systematic review (Figure 1).

### Study Characteristics

Multimedia Appendix 2 shows the study characteristics and patient demographic data of the included trials. Sample sizes ranged from 18 to 145, for a total of 894 patients with stroke. In total, 11 (47.8%) of the 23 trials were conducted in Korea [34,38,42-50], 3 (13%) in Portugal [51-53], 2 (8.7%) in Spain [54,55], 2 in China [36,37], 2 in Australia [56,57], and 1 (4.3%) each in Lithuania [58], Brazil [59] and Turkey [60].

Regarding the types of VR-based therapies, 16 (69.6%) trials administered VR as a singular session [36-38,44, 45,47-49,51,52,54-57,59,60], 5 (21.7%) trials were categorized as having used VR-based therapies plus occupational therapy [43,46,50,53,58], and 2 trials as having used VR-based therapies plus computer-assisted cognitive rehabilitation [34,42]. The frequency and duration of VR exposure varied considerably between trials. In our review, the duration of VR-based therapies ranged from 3 to 10 weeks, with the majority being 4 weeks, and the frequency of intervention varied from 2 to 5 times per week. The control group underwent conventional rehabilitation therapy [36-38,44,47,48,51,54,56,57,59] or other support interventions such as occupational therapy [43,45, 46,49,50,55,58], computer-assisted cognitive rehabilitation [34,42], and other rehabilitation therapies [52,53,60].

Of the 23 included trials, outcomes of our interest included global cognitive function (10 trials, 43.5%), executive function (5 trials, 21.7%), memory (5 trials), verbal fluency (2 trials, 8.7%), visuospatial ability (2 trials), attention (6 trials, 26.1%), depression symptom (5 trials), and the QoL (7 trials, 30.4%). Although the tools used for outcome evaluation varied across trials, all were valid scales, and the process of data collection was carried out by experienced staﬀ. Tools used to evaluate global cognitive function included the Montreal Cognitive Assessment (MoCA) [52,53,56,58], the Mini-Mental State Examination (MMSE) [34,45,49,51], and the Loewenstein Occupational Therapy Cognitive Assessment (LOTCA) [38,42]. Five trials evaluated the executive function domain using the CogState Groton Maze Learning Task [56], the Tower of London Test [34], the Stroop test [48], Trail-Making Test-B [51], and the the Digit Span Test [52]. Tools used to evaluate memory of patients with stroke included the visual recognition test [42], the visual span test [34], Wechsler Memory Scale-III [52], the Digit Span Test [54], and the Trail-Making Test [48]. The Digit Span Test [34] and Wechsler Memory Scale-III [52] were used to assess the verbal fluency of patients. Visuospatial ability was evaluated using the Motor-Free Visual Perception Test-3 [43] and the visual span test [34]. Attention was evaluated using the Single Letter Cancelation Test [53], Color of Color Word in Word–Color Test [34], and the Trail-Making Test [48,51,52,54]. The Hamilton Scale [36,46,55], the Beck Depression Inventory [37], and the Neurobehavioral Functioning Inventory [56] were used to assess the level of depression. The Stroke Impact Scale [38,47,50] was primarily used to assess the QoL, followed by the Short-Form 36 Health Survey Questionnaire [59], the Short-Form 8 Health Survey Questionnaire [44], the Nottingham Health Profile [60] and the EuroQoL Five Dimensions Questionnaire [57].

### Risk-of-Bias Assessment

**Figure 2:**
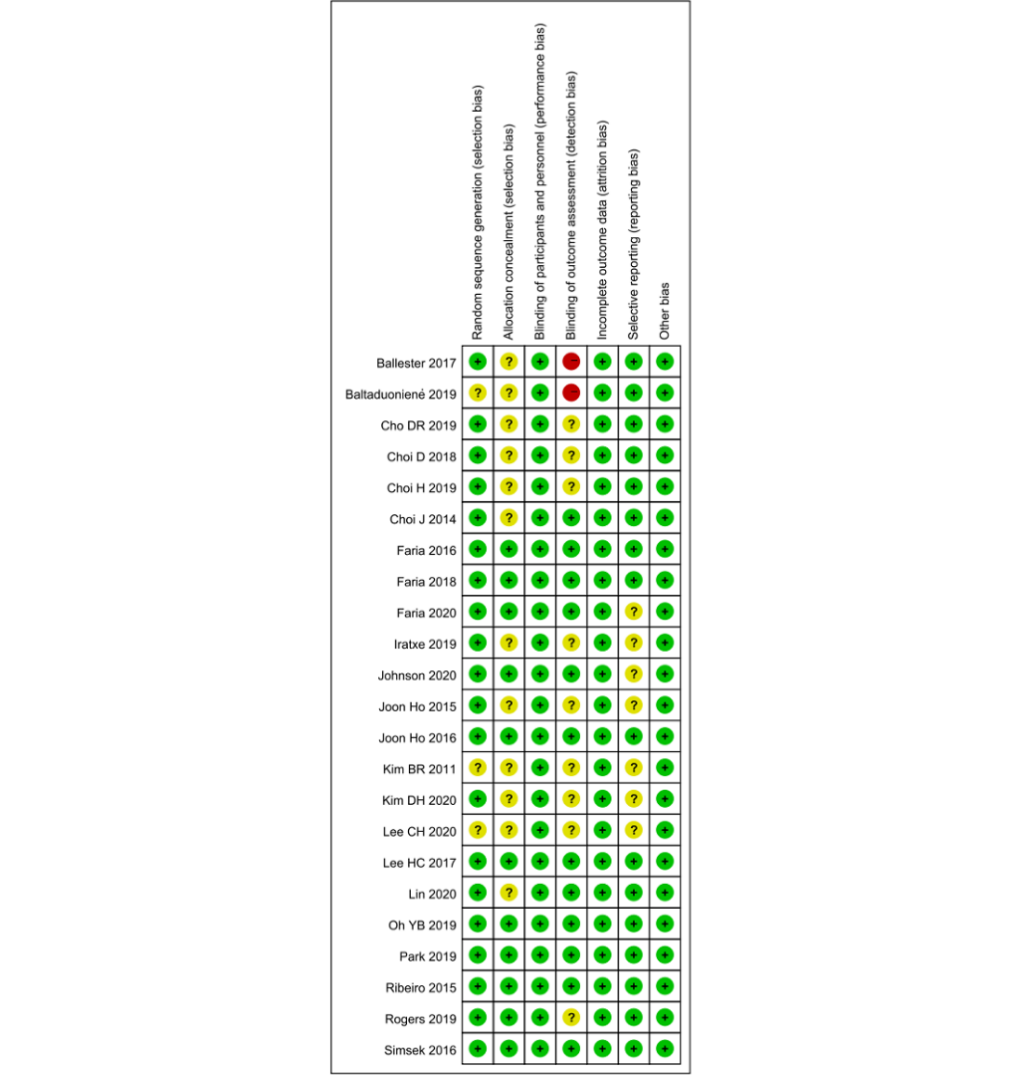
Risk-of-bias summary.

The risk of bias is summarized in Figure 2. In general, all 23 trials included in our review showed an acceptable risk of bias. The randomization sequence was adequately generated in 20 trials (86.9%), and 11 trials (47.8%) adequately concealed allocation. We categorized the risk of performance bias in all studies as low because blinding of participants and personnel was not possible in our systematic review. Approximately half of the trials blinded outcome assessors, and the risk of detection bias of these trials was judged as low. Of the 23 trials, 16 (69.6%) had no dropouts or used the intention-to-treat principle to compensate for dropouts; therefore, their risk of reporting bias was rated as low. All trials were categorized as having a low risk of bias in terms of attrition and other bias.

### Results of the Meta-analysis

The outcomes of global cognitive function, domain-specific cognition, and mental health were evaluated using various tools in the included trials. Domain-specific cognition included executive function, memory, verbal fluency, visuospatial ability, and attention in our review. Depression and the QoL were synthesized in our review. The change scores from baseline to final values were used in our final efficacy analysis. The results of our analysis of each outcome are as follows.

#### Global Cognitive Function

**Figure 3:**
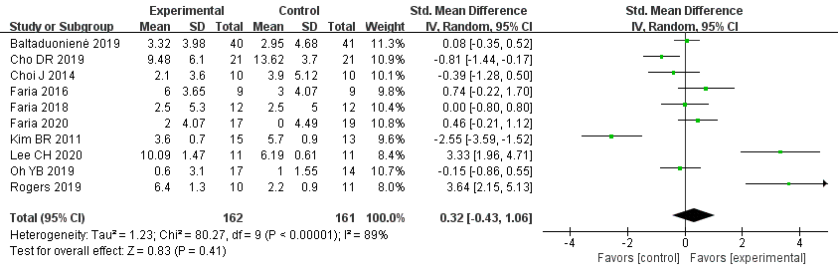
Forest plot for VR on global cognitive function. VR: virtual reality; CI: confidence interval.

As shown in Figure 3, effect sizes of global cognitive function could be generated for 10 trials [34,38,42,45,49,51-53,56,58] with 323 patients. A random-effects model was used as heterogeneity existed in our review (*I^2^*=89%, *P*<.001). Compared to the control, there was no evidence that VR-based therapies can significantly improve global cognitive function for patients with stroke (SMD=0.32, 95% CI=–0.43-1.06, *P*=.41).

In the subgroup that included VR versus the usual-care control group, the effect was significant (SMD=2.51, 95% CI=0.52-4.5, *P*=0.01; *I^2^*=87%, *P*<.001; high heterogeneity; N=61), while versus the other-support control group, no significant effect was detected (SMD=–0.41, 95% CI=–0.99-0.17, *P*=.16; *I^2^*=79%, *P*<.001; high heterogeneity; N=262). The subgroup interaction was significant (χ2=7.65, *P*=.01, *I^2^*=86.9%). In the subgroups of duration (less than 1-month or over 1-month intervention) and measurement instruments (MMSE, MoCA, or LOTCA), the subgroup interactions were nonsignificant (duration: χ2=0.12, *P*=.73, *I^2^*=0%; measurement instruments: χ2=2.83, *P*=.24, *I^2^*=29.2%), and heterogeneity was still significant in most of these subgroups.

#### Executive Function

**Figure 4:**
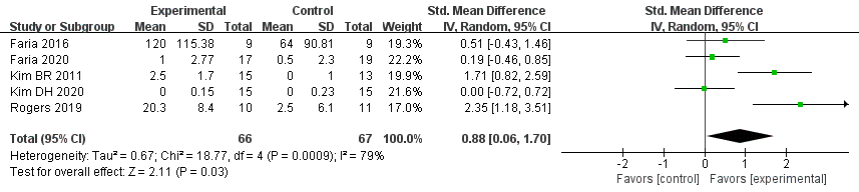
Forest plot for VR on executive function. VR: virtual reality; CI: confidence interval.

The effect of VR-based therapies on executive function was measured in 5 trials [34,48,51,52,56] including 133 patients. The pooled results with a random-effects model showed that VR-based therapies can significantly improve executive function compared to the control (SMD=0.88, 95% CI=0.06-1.70, *P*=.03), with high heterogeneity (*I^2^*=79%, *P*<.001; Figure 4). In the subgroups of the control groups (usual-care or other-support control) and duration (less than 1-month or over 1-month intervention), the subgroup interactions were nonsignificant (control: χ2=0.91, *P*=.34, *I^2^*=0%; duration: χ2=1.46, *P*=.23, *I^2^*=31.5%). Heterogeneity still existed in the subgroups.

#### Memory

**Figure 5:**
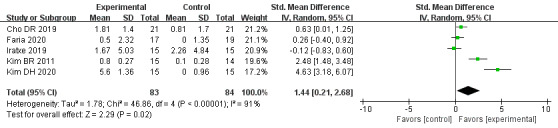
Forest plot for VR on memory. VR: virtual reality; CI: confidence interval.

The effect of VR-based therapies on memory was measured in 5 trials [34,42,48,52,54] involving 167 participants. Based on a random-effects model, our results showed a beneficial effect of VR-based therapies on enhancing memory in patients with stroke (SMD=1.44, 95% CI=0.21-2.68, *P*=.02), with high heterogeneity (*I^2^*=91%, *P*<.001; Figure 5). The control and duration subgroup interactions were nonsignificant (control: χ2=0.12, *P*=.72, *I^2^*=0%; duration: χ2=0.88, *P*=.35, *I^2^*=0%). Moderate heterogeneity existed in all subgroups.

#### Verbal Fluency

**Figure 6:**
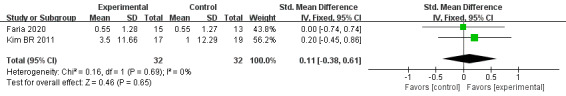
Forest plot for VR on verbal fluency. VR: virtual reality; CI: confidence interval.

The effect of VR-based therapies on verbal fluency was measured in 2 trials [34,52] involving 64 patients. Using a fixed-effects model, our meta-analysis showed no statistical significance on verbal fluency (SMD=0.11, 95% CI=–0.38-0.61, *P*=.65), with no heterogeneity (*I^2^*=0%, *P*=.65; Figure 6).

#### Visuospatial Ability

**Figure 7:**
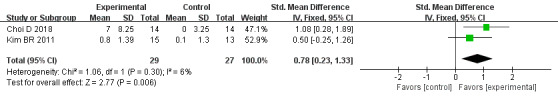
Forest plot for VR on visuospatial ability. VR: virtual reality; CI: confidence interval.

The effect of VR-based therapies on visuospatial ability was measured in 2 trials [34,43] involving 56 patients. We used a fixed-effects model for pooling the results, and the overall result showed that VR-based therapies have a significant effect on visuospatial ability compared with the control (SMD=0.78, 95% CI=0.23-1.33, *P*=.006), with no heterogeneity (*I^2^*=6%, *P*=.30; Figure 7).

#### Attention

**Figure 8:**
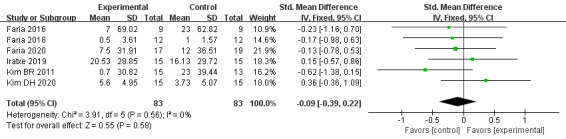
Forest plot for VR on attention. VR: virtual reality; CI: confidence interval.

The effect of VR-based therapies on attention was measured in 6 trials [34,48,51-54] involving 166 patients. We used a fixed-effects model for pooling the results, and the overall result presented no significant effect on attention between groups (SMD=–0.09, 95% CI=–0.39-0.22, *P*=.58), with no heterogeneity (*I^2^*=0%, *P*=.56; Figure 8).

#### Depression

The effect of VR-based therapies on depression symptoms was measured in 5 trials [36,38,46,55,56] including 255 participants. Lower scores indicated better psychological states. Based on a random-effects model, our results indicated that there was no statistical significance between the VR and control groups (SMD=0.20, 95% CI=–0.25-0.64, *P*=.39), with moderate heterogeneity (*I^2^*=56%, *P*=.06; Multimedia Appendix 3).

#### QoL

The effect of VR-based therapies on the QoL was measured in 7 trials [37,44,47,50,57,59,60] including 272 patients. Using a fixed-effects model, our results showed that VR-based therapies had no significant beneficial effect on the QoL (SMD=0.07, 95% CI=–0.17-0.31, *P*=.55), with low heterogeneity (*I^2^*=12%, *P*=.34; Multimedia Appendix 3).

### Publication Bias and Sensitivity Analyses

Funnel plots for global cognitive function showed symmetry. The Egger test of global cognitive function was not significant (*P*=.29, Multimedia Appendix 4), indicating no significant publication bias in our review. A sensitivity analysis was conducted, excluding individual trials one by one, to confirm the results of our meta-analysis. We found that except for the outcomes of executive function, the rest of the outcomes showed no substantial modification of the overall effect.

## Discussion

### Principal Findings

Our meta-analysis, based on 23 RCTs, indicated that VR therapy is an effective method of improving executive function, memory, and visuospatial ability on patients with stroke. However, current evidence fails to support the effects of VR-based therapies in improving global cognitive function, attention, verbal fluency, depression, and the QoL. Unlike prior systematic reviews [8,25] primarily focused more on either one type of VR, such as exercise-based VR, or one aspect of cognition indicators, such as only global cognitive function, our review [40] comprehensively evaluated the effect of VR-based therapies on global and domain-specific cognition and mental health outcomes.

VR-based therapies have demonstrated efficacy in the diagnosis, physical rehabilitation, and cognitive rehabilitation of individuals with neurocognitive disorders [17,19]. A growing body of studies has confirmed that the application of VR is effective in cognitive rehabilitation for patients affected by stroke [34,36,56]. However, our pooled results showed no significant benefits of VR in improving global cognitive function for patients with stroke. Our result was comparable to recently published meta-analysis reviews by Wiley et al [39] and Zhang et al [40], who reported that VR therapy is not superior to control interventions in improving global cognition in individuals with stroke. This result differs from another systematic review [25], which only included 4 studies published in 2018. The review found that there is a small-to-medium effect of VR therapy on cognitive outcomes for people after a stroke. A possible explanation for this conflicting finding is that most VR-based therapies do not always concentrate on the training of cognitive function. It remains to be determined whether more training sessions specifically focused on global cognitive function would affect outcomes.

Furthermore, the samples had relatively heterogeneous characteristics, such as stroke onset, the severity of the impairment, and lesion characteristics, which may affect cognition recovery in poststroke patients.

Cognitive function is a complex concept that includes various domain-specific cognition, such as attention, executive function, memory, and psychomotor speed [61]. In our review, we found improvements in executive function, memory, and visuospatial ability, indicating that VR can be considered an effective therapy for improving domain-specific cognition. These positive findings of VR therapy are in line with previous reviews [19,62,63], which report that VR represents a promising methodological approach, implementing specific cognitive and behavioural functions, such as executive function, attention, spatial cognition, memory, and language. A previous systematic review by Alexander et al [64] also supports these positive findings on domain-specific cognition. Wiley et al [39] employed a systematic review to examine the effectiveness of exercise-based VR therapy for poststroke, with 8 studies involving 196 participants, which did not find improvements in attention, memory, and language poststroke. The added value of VR in domain-specific cognition compared with most of the currently provided conventional therapies may be underlined by the following mechanisms. Flannery et al [65] stated that VR training activates brain metabolism, increases cerebral blood flow, and the release of neurotransmitters. Carrieri et al [66] confirmed that VR can foster the reactivation and improvement of various cortex functions and optimize the efficiency of the sensory cortex and is also effective in improving cognitive function.

Poor mental health after a stroke is common and complex. An individual’s QoL is greatly reduced by re-experiencing the situation with depression, anxiety, agitation, and stress. Many studies and clinical trials have shown the potential of VR in relieving stress, depression, and anxiety in an imagined space, which makes it possible to provide efficient educational and psychological training without causing harm to patients with psychological problems [36,67]. There are several possible explanations for these improvements in mental health. Damsbo [68] explained that the use of VR allows individuals to learn emotion regulation strategies in the context of life-like virtual environments, thereby alleviating their negative emotions during long-term recovery. Choi et al [44] thought VR, through quasi-naturalistic and realistic stimuli in a multisensory fashion, can significantly enhance patients’ awareness of the movement performed, as well as self-identification and self-recognition, and makes patients derive a high level of interest and enjoyment via the use of VR. However, the overall effects of VR on depression and the QoL were not encouraging in our meta-analysis. Some studies agree with the findings of our review [69,70]. The results of Lee [37] showed that some VR-based games are not suitable for patients following a stroke. Furthermore, patients with cognitive impairment may feel frustrated during the therapy, resulting in poor emotional experiences. Hence, the degree to which participants feel motivated and engaged during VR therapy can depend on the individual characteristics and the intervention content. The investigation of the mechanisms underlying how VR can influence mental functions is a critical point in stroke rehabilitation research. A likely cause of disagreement in findings on the QoL is that the QoL may worsen over time after stroke. However, the mean duration since stroke is inconsistent, even with no restrictions on duration among the trials included in our review. It is, therefore, possible that the duration since stroke is an important factor that should be considered when selecting a VR therapy to increase the QoL for stroke patients.

### Subgroup Analyses

Most subgroup analyses did not reveal significant differences between groups. Subgroup analyses consisted of a small number of comparisons, and the lack of relevant differences in most subgroup analyses might be caused by low statistical power. Only the subgroup analysis on global cognitive function showed that the effect of the usual-care control group was significant. There was also the issue with moderate-to-high heterogeneity in our meta-analysis, and grouping the included trials by measurement tools, duration of intervention, and delivery of control did not eliminate heterogeneity. Given the highly variable interventions of included trials, it is not surprising to find high heterogeneity in this case.

### Strengths and Limitations

This meta-analysis followed the guidelines for performing rigorous systematic reviews [41]. Our review was only based on RCTs, which reinforces the evidence of our results. We also proposed a rigorous screening and search strategy to identify the most comprehensive literature in five major databases. Consequently, the results of our review are a widespread belief. However, there were still some potential limitations to our review. First, the number of trials included in our meta-analysis was limited. Hence, the power to detect small effect sizes was limited. Second, the included trials were extremely varied in terms of intervention contents and doses, and delivery of the control and measurement instruments, which made it rather complex to determine the optimal intensity of VR programs. Third, the quality, quantity, and sample size of included trials were far from ideal. Concealed treatment allocation was often not guaranteed, and blinding of outcome assessments was often not carried out. For example, only less than half of the included trials reported details of allocation concealment, leading to selection bias or confounding of the pooled results. Concerning detecting bias, almost half of the included trials failed to report information or had a high risk of blinding outcome assessment. Despite the rigorous nature of the included research designs (only included RCTs), our results must be interpreted with caution. Fourth, since we only considered effect post-intervention, the sustainability of VR-based therapy effects was not explored in our review. Indeed, details of long-term effects were unavailable in most trials included. Further research is needed to assess whether the effect observed in our review would persist over time and, if not, to evaluate at what duration and frequency the VR therapy should be repeated in order to sustain its effect. Finally, although a significant amount of work has been done in this area with promising results, the relevant characteristics of VR systems and the quantification of their impact on recovery are not yet clearly understood. As a result, we do not know how the different parameters of the proposed VR scenarios exactly affect recovery or whether they are effective at all. There is also a need to consider individual variability in order to optimize the impact of training.

### Implications for Clinical Practice and Future Research

Notwithstanding potential limitations, the present findings offer some implications for researchers and health practitioners. In this high-tech era, clinicians may have more options and alternatives by providing an interactive and visually stimulating approach for patients with stroke, especially for those who cannot easily access traditional rehabilitation methods. The following issues need to be considered when applying VR-based therapies. First, VR should be adapted to the patient’s needs and characteristics in performing activities, tasks, and tests. Second, safety problems need to be highlighted for older adults with reduced vision or other sensory problems. It is critical to consider a methodology type and an interaction technique that will result in the safe implementation of a VR therapy for patients with sensory and cognitive impairment. Finally, the price of VR equipment should be considered to meet the needs of target populations. In addition, there is a need for further research in this field to promote cost-effective care.

### Conclusions

VR is a promising approach and can be used effectively in clinical neurorehabilitation. Although existing studies are limited, this review demonstrated statistically significant effects of VR-based therapies on executive function, memory, and visuospatial function in patients with stroke, but not on global cognitive function, attention, verbal fluency, depression, and the QoL. Larger, multicenter RCTs are warranted to confirm these positive effects. The completion of high‐quality trials will ultimately advance the knowledge about optimal cognitive and psychological rehabilitation strategies for patients with stroke.

## Appendix

Multimedia Appendix 1 Search strategy.

Multimedia Appendix 2 Study characteristics.

Multimedia Appendix 3 Forest plot for VR on depression and QoL. VR: virtual reality; QoL: quality of life.

Multimedia Appendix 4 The Egger funnel plot of publication bias.

## Abbreviations

CI: confidence interval
LOTCA: Loewenstein Occupational Therapy Cognitive Assessment
MMSE: Mini-Mental State Examination
MoCA: Montreal Cognitive Assessment
QoL: quality of life
RCT: randomized controlled trial
SMD: standardized mean difference
VR: virtual reality
WMD: weighted mean difference
