# Supplementary material for: Impact of Virtual Reality-Based Therapies on Cognition and Mental Health of Stroke Patients: Systematic Review and Meta-analysis
Source: J Med Internet Res. 2021 Nov 17;23(11):e31007. doi: 10.2196/31007 (PMC8663637; doi:10.2196/31007)
Supplement: Multimedia Appendix 1 [file jmir_v23i11e31007_app1.docx]

**Search Strategy.**

Pubmed

| Disease: stroke | |
| --- | --- |
| #1 | "Stroke"[Mesh] OR Stroke* OR "Cerebrovascular Accident*" OR CVA OR CVAS OR "Cerebrovascular Apoplexy" OR "Apoplexy, Cerebrovascular" OR "Vascular Accident, Brain" OR "Brain Vascular Accident*" OR " Cerebrovascular Stroke*" OR Apoplexy OR "Cerebral Stroke*" OR “stroke unit” OR “stroke patient*” |
| #2 | "Cerebrovascular Disorders"[Mesh] OR "Cerebrovascular Disorder*" OR "Intracranial Vascular Disease*" OR "Brain Vascular Disorder*" OR "brain injur*" OR "acquired brain injur*" |
| #3 | "Subarachnoid Hemorrhage"[Mesh] OR " intracranial hemorrhage" OR SAH |
| #4 | #1 OR #2 OR #3 |
| Intervention:VR | |
| #5 | "Virtual Reality Exposure Therapy"[Mesh] OR "virtual real*" OR VR OR "virtual-real*" OR "virtual reality therap*" |
| #6 | "Video gam*" OR "computer gam*" OR "gaming consol*" OR "interactive gam*" OR xbox OR playstation OR nintendo |
| #7 | Virtual NEAR/5 (environment* or object* or world* or treatment* or system* or program* or rehabilitation* or therap*) |
| #8 | #5 OR #6 OR #7 |
| #9 | #4 AND #8 |

Embase

| Disease: stroke | |
| --- | --- |
| #1 | 'cerebrovascular accident'/exp OR stroke* OR 'cerebrovascular accident*' OR cva OR cvas OR 'cerebrovascular apoplexy' OR 'vascular accident, brain' OR 'brain vascular accident*' OR 'cerebrovascular stroke*' OR apoplexy OR 'cerebral stroke*' OR 'stroke unit or stroke patient*' |
| #2 | (cereb* OR brain* OR vertebrobasilar) NEAR/5 (infarct* OR isch?emi* OR thrombo* OR emboli* OR apoplexy) |
| #3 | (cerebral OR brain OR subarachnoid) NEAR/5 (haemorrhage OR hemorrhage OR haematoma OR hematoma OR bleed*) |
| #4 | 'hemiplegia'/exp OR hemiplegia* OR hemiparesis OR paresis |
| #5 | #1 OR #2 OR #3 OR #4 |
| Intervention：VR | |
| #6 | 'virtual reality'/exp OR 'virtual real*' OR vr OR 'virtual-real*' OR 'virtual reality therap*' |
| #7 | 'video gam*' OR 'computer gam*' OR 'gaming consol*' OR 'interactive gam*' OR xbox OR playstation OR nintendo |
| #8 | virtual NEAR/5 (environment* OR object* OR world* OR treatment* OR system* OR program* OR rehabilitation* OR therap*) |
| #9 | #6 OR #7 OR #8 |
| #10 | #5 AND #9 |

MEDLINE（ovid）

| Disease: stroke |
| --- |
| 1 Cerebral Ischemia/ or exp. Cerebrovascular Accidents/ or exp Ischemia/ or exp Cardiovascular Disorders/ or Stroke.mp. |
| 2 Cerebrovascular disease/ or exp basal ganglion hemorrhage/ or exp brain hematoma/ or exp brain hemorrhage/ or exp brain infarction/ or exp brain ischemia/ or exp carotid artery disease/ or cerebral artery disease/ or exp cerebrovascular malformation/ or exp intracranial aneurysm/ or exp occlusive cerebrovascular disease/ or stroke/ or stroke unit/ or stroke patient/ |
| 3 (Hemorrhag* or Subarachnoid Hemorrhage or intracranial hemorrhage).mp. |
| 4 (Poststroke or post-stroke or cerebrovasc* or cerebral vascular or brain vasc* or cerebral vasc* or cva* or apoplexy* or SAH).mp. |
| 5 (Brain* or cerebr* or cerebell* or intracran* or intracerebral N5 isch?emi* or infarct* or thrombo* or emboli* or occlus*).mp. |
| 6 1 or 2 or 3 or 4 or 5 |
| Intervention:VR |
| 7 (Virtual real* or virtual-real* or VR).mp. |
| 8 (Augment* reality or virtual reality or augment* gam* or virtual gam*).mp. |
| 9 (Video gam* or computer gam* or gaming consol* or interactive gam* or Nintendo Wii or kinect or nintendo or playstation or xbox or gam* program).mp. |
| 10 (Virtual adj3 (environment* or object* or world* or treatment* or system* or program* or rehabilitation* or therap*)).mp. |
| 11 7 or 8 or 9 or 10 |
| 12 6 and 11 |

Cochrane Library

| Disease: stroke | |
| --- | --- |
| #1 | MeSH descriptor: [Stroke] explode all tress |
| #2 | MeSH descriptor: [Subarachnoid Hemorrhage] explode all tress |
| #3 | MeSH descriptor: [Hemiplegia] explode all tress |
| #4 | (Stroke* OR "Cerebrovascular Accident*" OR CVA OR CVAS OR "Cerebrovascular Apoplexy" OR "Apoplexy, Cerebrovascular" OR "Vascular Accident, Brain" OR "Brain Vascular Accident*" OR " Cerebrovascular Stroke*" OR Apoplexy OR "Cerebral Stroke*" OR “stroke unit” OR “stroke patient*”):ti,ab,kw |
| #5 | "Cerebrovascular Disorder*" OR "Intracranial Vascular Disease*" OR "Brain Vascular Disorder*" OR "brain injur*" OR "acquired brain injur*" |
| #6 | " intracranial hemorrhage" OR SAH OR hemiplegia* OR hemiparesis OR paresis |
| #7 | #1 OR #2 OR #3 OR #4 OR #5 OR #6 |
| Intervention:VR | |
| #8 | MeSH descriptor: [Virtual Reality Exposure Therapy] explode all trees |
| #9 | "virtual real*" OR VR OR "virtual-real*" OR "virtual reality therap*" |
| #10 | "Video gam*" OR "computer gam*" OR "gaming consol*" OR "interactive gam*" OR xbox OR playstation OR nintendo |
| #11 | Virtual NEAR/5 (environment* or object* or world* or treatment* or system* or program* or rehabilitation* or therap*) |
| #12 | #8 OR #9 OR #10 OR #11 |
| #13 | #7 AND #12 |

APA Psyclnfo

| Disease: stroke | |
| --- | --- |
| S1 | Stroke* OR "Cerebrovascular Accident*" OR CVA OR CVAS OR "Cerebrovascular Apoplexy" OR "Apoplexy, Cerebrovascular" OR "Vascular Accident, Brain" OR "Brain Vascular Accident*" OR " Cerebrovascular Stroke*" OR Apoplexy OR "Cerebral Stroke*" OR “stroke unit” OR “stroke patient*” |
| S2 | "Cerebrovascular Disorder*" OR "Intracranial Vascular Disease*" OR "Brain Vascular Disorder*" OR "brain injur*" OR "acquired brain injur*" |
| S3 | " intracranial hemorrhage" OR SAH OR hemiplegia* OR hemiparesis OR paresis |
| S4 | S1 OR S2 OR S3 |
| Intervention:VR | |
| S5 | "Virtual Reality Exposure Therapy" OR "virtual real*" OR VR OR "virtual-real*" OR "virtual reality therap*" |
| S6 | "Video gam*" OR "computer gam*" OR "gaming consol*" OR "interactive gam*" OR xbox OR playstation OR nintendo |
| S7 | Virtual NEAR/5 (environment* or object* or world* or treatment* or system* or program* or rehabilitation* or therap*) |
| S8 | S5 OR S6 OR S7 |
| S9 | S4 AND S8 |
