# Supplementary material for: Impact of Virtual Reality-Based Therapies on Cognition and Mental Health of Stroke Patients: Systematic Review and Meta-analysis
Source: J Med Internet Res. 2021 Nov 17;23(11):e31007. doi: 10.2196/31007 (PMC8663637; doi:10.2196/31007)
Supplement: Multimedia Appendix 2 [file jmir_v23i11e31007_app2.docx]

| **Study/Year** | **Mean age** | **Number** | **Intervention** | **Control** | **Setting** | **Measure** | **Effect size and Confidence interval (CI)** |
| --- | --- | --- | --- | --- | --- | --- | --- |
| **Global cognitive function** | | | | | | | |
| Baltaduonienė/2019 | T: 69.71±11.67  C:74.33±10.27 | T:40  C:40 | **Type：**Individual OT + practised VE rehabilitation system activities (SeeMeᴿ Brontes Processing, Poland)  **Frequency:**  45 min/time, 2 times/ week, 32 days+  45 min/time, 3 times/ week, 32 days | **Type：**Individual OT  **Frequency:**  45 min/time, 5 times/ week, 32 days | Lithuania | MoCA | 0.08 [-0.35, 0.52] |
| Cho DR/2019 | T:58.43±14.61  C:54.71±16.23 | T:21  C:21 | **Type：**Virtual reality training + computerized neurocognitive training  **Frequency:**  30min/time, 5 times/week, 4 weeks +  30min/time, 5 times/week, 4 weeks | **Type：**Computerized neurocognitive training  **Frequency:**  60min/time, 5 times/week, 4 weeks | Korea | LOTCA | -0.81 [-1.44, -0.17] |
| Choi J/2014 | T:64.30±10.3  C:64.70±11.3 | T:10  C:10 | **Type：**Commercial gaming-based VR movement therapy using the Wii (Nintendo)  **Frequency:**  30 min/time, 5 times/week, 4 weeks | **Type：**OT  **Frequency:**  30 min/time, 5 times/week, 4 weeks | Korea | MMSE | -0.39 [-1.28, 0.50] |
| Faria /2016 | T:58 (48-71)  C:53 (50.5-65.5) | T:9  C:9 | **Type：**Cognitive training using a virtual simulation of a city (the Reh@City)  **Frequency:**  20 min/time, 2-3times/week, 4-6 weeks | **Type：**Conventional rehabilitation therapy  **Frequency:**  20 min/time, 2-3 times/week, 4-6 weeks | Portugal | MMSE | 0.74 [-0.22, 1.70] |
| Faria /2018 | T:57.1±11.0  C:68.9±9.8 | T:12  C:12 | **Type：**Reh@Task + conventional OT  **Frequency:**  45 min/time, 3 times/week, 4 weeks | **Type：**Spatial and time orientation activities, and writing training  + conventional OT  **Frequency:**  45-60 min/time, 3 times/week, 4 weeks | Portugal | MoCA | 0.00 [-0.80, 0.80] |
| Faria /2020 | T:59.14±11.81  C:65.00±6.20 | T:17  C:19 | **Type：**Paper-and-pencil tasks using the Reh@City v2.0  **Frequency:**  90 min/time, 3 times/week, 8 weeks | **Type：**Paper-and-pencil intervention using the task generator  **Frequency:**  45 min/time, 3 times/week, 8 weeks | Portugal | MoCA | 0.46 [-0.21, 1.12] |
| Kim BR/2011 | T:66.5±11.0  C:62.0±15.8 | T:15  C:13 | **Type：**Virtual reality training using IREX system+ computer-assisted cognitive rehabilitation  **Frequency:**  30 min/time, 3 times/week, 4 weeks+  30 min/time, twice/week, 4 weeks | **Type：**Computer-assisted cognitive rehabilitation  **Frequency:**  30 min/time, 5 times/week, 4 weeks | Korea | MMSE | -2.55 [-3.59, -1.52] |
| Lee CH/2020 | T:58.18±8.22  C:59.09±11.65 | T:11  C:11 | **Type：**Virtual reality cognitive training  **Frequency:**  30 min/time, 5 times/week, 4 weeks | **Type：**Conventional rehabilitation therapy  **Frequency:**  30 min/time, 5 times/week, 4 weeks | Korea | LOTCA | 3.33 [1.96, 4.71] |
| Oh YB/2019 | T:57.4±12.2  C:52.6±10.7 | T:17  C:14 | **Type:** Real instrument training in a VR environment  **Frequency:**  30 min/time, 3 times/week, 6 weeks | **Type:** Conventional OT  **Frequency:**  30 min/time, 3 times/week, 6 weeks | Korea | MMSE | -0.15 [-0.86, 0.55] |
| Rogers/2019 | T:64.3±17.4  C:64.6±12.0 | T:10  C:11 | **Type：**Virtual rehabilitation using the Elements VR interactive tabletop system  **Frequency:**  30-40 min/time, 3 times/week, 4 weeks | **Type：**Conventional rehabilitation therapy  **Frequency:**  30-40 min/time, 3 times/week, 4 weeks | Australia | MoCA | 3.64 [2.15, 5.13] |
| **Executive function** | | | | | | | |
| Faria /2016 | T:58 (48-71)  C:53 (50.5-65.5) | T:9  C:9 | **Type：**Cognitive training using a virtual simulation of a city (the Reh@City)  **Frequency:**  20 min/time, 2-3times/week, 4-6 weeks | **Type：**Conventional rehabilitation therapy  **Frequency:**  20 min/time, 2-3 times/week, 4-6 weeks | Portugal | TMT-B | 0.51 [-0.43, 1.46] |
| Faria /2020 | T:59.14±11.81  C:65.00±6.20 | T:17  C:19 | **Type：**Paper-and-pencil tasks using the Reh@City v2.0  **Frequency:**  90 min/time, 3 times/week, 8 weeks | **Type：**Paper-and-pencil intervention using the task generator  **Frequency:**  45 min/time, 3 times/week, 8 weeks | Portugal | DST | 0.19 [-0.46, 0.85] |
| Kim BR/2011 | T:66.5±11.0  C:62.0±15.8 | T:15  C:13 | **Type：**Virtual reality training using IREX system+ computer-assisted cognitive rehabilitation  **Frequency:**  30 min/time, 3 times/week, 4 weeks+  30 min/time, twice/week, 4 weeks | **Type：**Computer-assisted cognitive rehabilitation  **Frequency:**  30 min/time, 5 times/week, 4 weeks | Korea | TOL | 1.71 [0.82, 2.59] |
| Kim DH/2020 | T:59.4±1.8  C:54.73±2.98 | T:15  C:15 | **Type：**Virtual reality training using RAPAEL Smart Glove™  **Frequency:**  60 min/time, 3 times/week, 8 weeks | **Type：**Conventional rehabilitation therapy  **Frequency:**  60 min/time, 3 times/week, 8 weeks | Korea | ST | 0.00 [-0.72, 0.72] |
| Rogers/2019 | T:64.3±17.4  C:64.6±12.0 | T:10  C:11 | **Type：**Virtual rehabilitation using the Elements VR interactive tabletop system  **Frequency:**  30-40 min/time, 3 times/week, 4 weeks | **Type：**Conventional rehabilitation therapy  **Frequency:**  30-40 min/time, 3 times/week, 4 weeks | Australia | GMLT | 2.35 [1.18, 3.51] |
| **Memory** | | | | | | | |
| Cho DR/2019 | T:58.43±14.61  C:54.71±16.23 | T:21  C:21 | **Type：**Virtual reality training + computerized neurocognitive training  **Frequency:**  30min/time, 5 times/week, 4 weeks +  30min/time, 5 times/week, 4 weeks | **Type：**Computerized neurocognitive training  **Frequency:**  60min/time, 5 times/week, 4 weeks | Korea | VRT-recall | 0.63 [0.01, 1.25] |
| Faria /2020 | T:59.14±11.81  C:65.00±6.20 | T:17  C:19 | **Type：**Paper-and-pencil tasks using the Reh@City v2.0  **Frequency:**  90 min/time, 3 times/week, 8 weeks | **Type：**Paper-and-pencil intervention using the task generator  **Frequency:**  45 min/time, 3 times/week, 8 weeks | Portugal | memory from WMS-III | 0.26 [-0.40, 0.92] |
| Iratxe/2019 | T:58.43±15.71  C:71.55±11.37 | T:15  C:15 | **Type：**Physical activity using with the Nintendo Wii Sports Resort  **Frequency:**  30 min/time, 3 times/week, 8 weeks | **Type：**Conventional rehabilitation therapy  **Frequency:**  30 min/time, 3 times/week, 8 weeks | Spain | DST | -0.12 [-0.83, 0.60] |
| Kim BR/2011 | T:66.5±11.0  C:62.0±15.8 | T:15  C:13 | **Type：**Virtual reality training using IREX system+ computer-assisted cognitive rehabilitation  **Frequency:**  30 min/time, 3 times/week, 4 weeks+  30 min/time, twice/week, 4 weeks | **Type：**Computer-assisted cognitive rehabilitation  **Frequency:**  30 min/time, 5 times/week, 4 weeks | Korea | VST | 2.48 [1.48, 3.48] |
| Kim DH/2020 | T:59.4±1.8  C:54.73±2.98 | T:15  C:15 | **Type：**Virtual reality training using RAPAEL Smart Glove™  **Frequency:**  60 min/time, 3 times/week, 8 weeks | **Type：**Conventional rehabilitation therapy  **Frequency:**  60 min/time, 3 times/week, 8 weeks | Korea | TMT | 4.63 [3.18, 6.07] |
| **Verbal fluence** | | | | | | | |
| Faria /2020 | T:59.14±11.81  C:65.00±6.20 | T:17  C:19 | **Type：**Paper-and-pencil tasks using the Reh@City v2.0  **Frequency:**  90 min/time, 3 times/week, 8 weeks | **Type：**Paper-and-pencil intervention using the task generator  **Frequency:**  45 min/time, 3 times/week, 8 weeks | Portugal | vocabulary from WAIS-III | 0.00 [-0.74, 0.74] |
| Kim BR/2011 | T:66.5±11.0  C:62.0±15.8 | T:15  C:13 | **Type：**Virtual reality training using IREX system+ computer-assisted cognitive rehabilitation  **Frequency:**  30 min/time, 3 times/week, 4 weeks+  30 min/time, twice/week, 4 weeks | **Type：**Computer-assisted cognitive rehabilitation  **Frequency:**  30 min/time, 5 times/week, 4 weeks | Korea | DST | 0.20 [-0.45, 0.86] |
| **Visuospatial ability** | | | | | | | |
| Choi D/2018 | T:49.50±23.00 C:51.00±13.75 | T:14  C:14 | **Type：**Wii Fit virtual reality training + conventional physical and OT  **Frequency:**  30 min/time, 3 times/week, 6 weeks+  90 min/time, 5 times/week, 6 weeks | **Type：**General balance training + conventional physical and OT  **Frequency:**  30 min/time, 3 times/week, 6 weeks+  90 min/time, 5 times/week, 6 weeks | Korea | MVPT-3 | 1.08 [0.28, 1.89] |
| Kim BR/2011 | T:66.5±11.0  C:62.0±15.8 | T:15  C:13 | **Type：**Virtual reality training using IREX system+ computer-assisted cognitive rehabilitation  **Frequency:**  30 min/time, 3 times/week, 4 weeks+  30 min/time, twice/week, 4 weeks | **Type：**Computer-assisted cognitive rehabilitation  **Frequency:**  30 min/time, 5 times/week, 4 weeks | Korea | VST | 0.50 [-0.25, 1.26] |
| **Attention** | | | | | | | |
| Faria /2016 | T:58 (48-71)  C:53 (50.5-65.5) | T:9  C:9 | **Type：**Cognitive training using a virtual simulation of a city (the Reh@City)  **Frequency:**  20 min/time, 2-3times/week, 4-6 weeks | **Type：**Conventional rehabilitation therapy  **Frequency:**  20 min/time, 2-3 times/week, 4-6 weeks | Portugal | TMT-A | -0.23 [-1.16, 0.70] |
| Faria /2018 | T:57.1±11.0  C:68.9±9.8 | T:12  C:12 | **Type：**Reh@Task + conventional OT  **Frequency:**  45 min/time, 3 times/week, 4 weeks | **Type：**Spatial and time orientation activities, and writing training  + conventional OT  **Frequency:**  45-60 min/time, 3 times/week, 4 weeks | Portugal | SLCT | -0.17 [-0.98, 0.63] |
| Faria /2020 | T:59.14±11.81  C:65.00±6.20 | T:17  C:19 | **Type：**Paper-and-pencil tasks using the Reh@City v2.0  **Frequency:**  90 min/time, 3 times/week, 8 weeks | **Type：**Paper-and-pencil intervention using the task generator  **Frequency:**  45 min/time, 3 times/week, 8 weeks | Portugal | TMT-A | -0.13 [-0.78, 0.53] |
| Iratxe/2019 | T:58.43±15.71  C:71.55±11.37 | T:15  C:15 | **Type：**Physical activity using with the Nintendo Wii Sports Resort  **Frequency:**  30 min/time, 3 times/week, 8 weeks | **Type：**Conventional rehabilitation therapy  **Frequency:**  30 min/time, 3 times/week, 8 weeks | Spain | TMT-A | 0.15 [-0.57, 0.86] |
| Kim BR/2011 | T:66.5±11.0  C:62.0±15.8 | T:15  C:13 | **Type：**Virtual reality training using IREX system+ computer-assisted cognitive rehabilitation  **Frequency:**  30 min/time, 3 times/week, 4 weeks+  30 min/time, twice/week, 4 weeks | **Type：**Computer-assisted cognitive rehabilitation  **Frequency:**  30 min/time, 5 times/week, 4 weeks | Korea | CCWT | -0.62 [-1.38, 0.15] |
| Kim DH/2020 | T:59.4±1.8  C:54.73±2.98 | T:15  C:15 | **Type：**Virtual reality training using RAPAEL Smart Glove™  **Frequency:**  60 min/time, 3 times/week, 8 weeks | **Type：**Conventional rehabilitation therapy  **Frequency:**  60 min/time, 3 times/week, 8 weeks | Korea | TMT | 0.36 [-0.36, 1.09] |
| **Depression** | | | | | | | |
| Ballester/2017 | T:65.03±10.33  C:61.75±12.94 | T:17  C:18 | **Type：**Home-based training paradigm based on the Spheroids scenario  **Frequency:**  20-60min/time, 5 times/week, 3 weeks | **Type：**Domiciliary OT  **Frequency:**  20-60min/time, 5 times/week, 3 weeks | Spain | HS | -0.36 [-1.03, 0.31] |
| Joon Ho/2015 | T:53.3±11.8  C:54.6±13.4 | T:16  C:16 | **Type：**Game-based VR rehabilitation with the RehabMaster™ system +OT  **Frequency:**  60 min/time, 5 times/week, 4 weeks | **Type：**OT  **Frequency:**  60 min/time, 5 times/week, 4 weeks | Korea | HS-D | -0.20 [-0.90, 0.49] |
| Lee CH/2020 | T:58.18±8.22  C:59.09±11.65 | T:11  C:11 | **Type：**Virtual reality cognitive training  **Frequency:**  30 min/time, 5 times/week, 4 weeks | **Type：**Conventional rehabilitation therapy  **Frequency:**  30 min/time, 5 times/week, 4 weeks | Korea | BDI | 0.13 [-0.71, 0.97] |
| Lin/2020[33] | T:64.5±13.5  C:66.9±13.3 | T:38  C:107 | **Type:** VR training using wireless VR device and Kinect sensor  **Frequency:**  30 min/time, 5 times/week, 4 weeks | **Type：**Conventional rehabilitation therapy  **Frequency:**  60 min/time, 5 times/week, 4 weeks | China | HS-D | 0.58 [0.21, 0.96] |
| Rogers/2019 | T:64.3±17.4  C:64.6±12.0 | T:10  C:11 | **Type：**Virtual rehabilitation using the Elements VR interactive tabletop system  **Frequency:**  30-40 min/time, 3 times/week, 4 weeks | **Type：**Conventional rehabilitation therapy  **Frequency:**  30-40 min/time, 3 times/week, 4 weeks | Australia | NFI-D | 0.78 [-0.12, 1.67] |
| **Quality of life** | | | | | | | |
| Choi H/2019 | T:58.00±15.15  C:59.33±13.63 | T:12  C:12 | **Type：**Gesture Recognition mirror therapy using a Leap motion controller  (Leap Motion, Inc., USA)  **Frequency:**  30 min/time, 3 times/week, 5 weeks | **Type：**Sham therapy  **Frequency:**  30 min/time, 3 times/week, 5 weeks | Korea | SF-8 | 0.52 [-0.30, 1.33] |
| Johnson/2020 | T:64.71±3.9  C:59.31±5.6 | T:28  C:30 | **Type：**Exercise training using Jintronix Rehabilitation System and Microsoft Xbox Kinect camera  **Frequency:**  45 min/time, twice/week, 8 weeks | **Type：**Conventional rehabilitation therapy  **Frequency:**  45 min/time, twice/week, 8 weeks | Australia | EQ-5D | 0.00 [-0.52, 0.52] |
| Joon Ho/2016 | T:57.2±10.3  C:59.8±13.0 | T:24  C:22 | **Type：**Taring using the RAPAEL Smart Glove™ biofeedback system  **Frequency:**  60 min/time, 5 times/week, 4 weeks | **Type：**Conventional rehabilitation therapy  **Frequency:**  60 min/time, 5 times/week, 4 weeks | Korea | SIS | -0.14 [-0.72, 0.44] |
| Lee HC/2017 | T:59.35±8.95  C:55.76± 9.59 | T:26  C:21 | **Type：**Interactive VR balance-related games using a television and a commercial game+ conventional rehabilitation therapy  **Frequency:**  45 min/time, twice/week, 6 weeks+  45 min/time, twice/week, 6 weeks | **Type：**Conventional rehabilitation therapy  **Frequency:**  90 min/time, twice/week, 6 weeks | Taiwan，China | SIS | 0.03 [-0.54, 0.61] |
| Park/2019 | T:53.5±13.0  C:51.5±16.7 | T:12  C:13 | **Type：**VR training using the Rapael Smart Board™ + conventional OT  **Frequency:**  30 min/time, 5 times/week, 4 weeks+  30 min/time, 5 times/week, 4 weeks | **Type：**Conventional OT  **Frequency:**  60 min/time, 5 times/week, 4 weeks | Korea | SIS | 0.97 [0.13, 1.80] |
| Ribeiro/2015 | T:53.7±6.1  C:52.8±8.6 | T:15  C:15 | **Type：**Virtual rehabilitation using the NintendoH Wii  **Frequency:**  60 min/time, twice/week, 8 weeks | **Type：**Conventional rehabilitation therapy  **Frequency:**  60 min/time, twice/week, 8 weeks | Brazil | SF-36 | -0.16 [-0.88, 0.56] |
| Şimşek/2016 | T:54.15±20.29  C:61.5±11.63 | T:20  C:22 | **Type：**Virtual rehabilitation using the N-Wii play systems  **Frequency:**  45-60 min/time, 3 times/week, 10 weeks | **Type：**Bobath neurodevelopmental treatment  **Frequency:**  45-60 min/time, 3 times/week, 10 weeks | Turkey | NHP | -0.10 [-0.71, 0.50] |

I: Intervention Group; C: Control Group; OT: Occupational Therapy; VE: Virtual Environment; MMSE: Mini-mental State Examination; MOCA: Montreal Cognitive Assessment Scale; LOTCA: Loewenstein Occupational Therapy Cognitive Assessment; VRT: Visual Recognition Test; MVPT-3: Motor-free Visual Perception Test-3; SF-8: Short Form 8 Health Survey Questionnaire; TMT: Trail Making Test; SLCT: Single Letter Cancelation Test; DST: Digit Span Test; WMS-III: Wechsler Memory Scale-III; EQ-5D: EuroQol Five Dimensions Questionnaire; SIS: Stroke Impact Scale; TOL: Tower of London Test; VST: Visual Span Test; CCWT: Color of Color Word in Word-Color Test; ST: Stroop Test; HS: Hamilton Scale; BDI: Beck Depression Inventory; SF-36: Short Form 36 Health Survey Questionnaire; GMLT: CogState Groton Maze Learning Task; NFI: Neurobehavioral Functioning Inventory; NHP: Nottingham Health Profile.
