# Supplementary figures and images for: Impact of Virtual Reality-Based Therapies on Cognition and Mental Health of Stroke Patients: Systematic Review and Meta-analysis
Source: J Med Internet Res. 2021 Nov 17;23(11):e31007. doi: 10.2196/31007 (PMC8663637; doi:10.2196/31007)

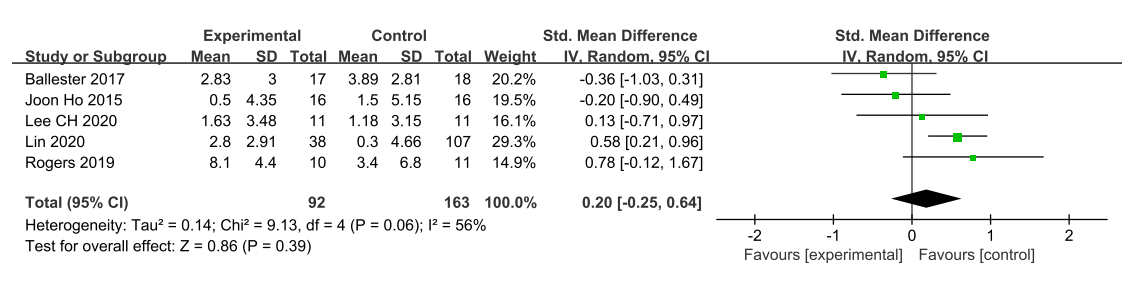


Forest plot for VR on depression.


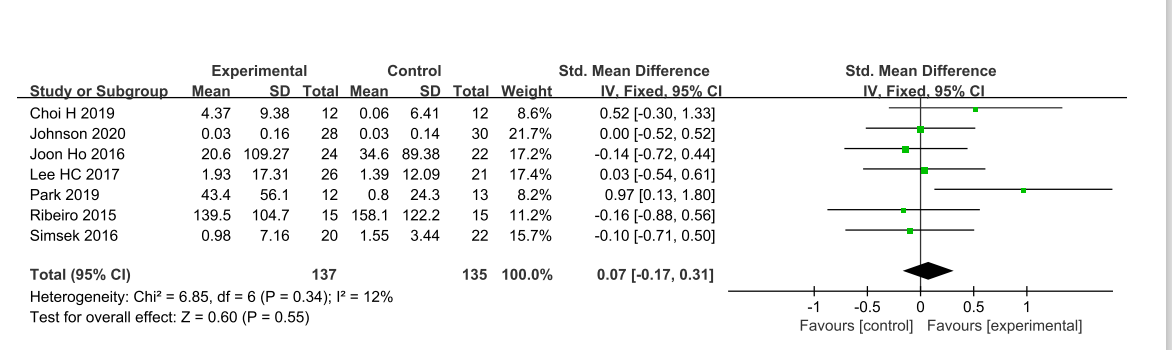


Forest plot for VR on quality of life.

Supplement: Multimedia Appendix 3 [file jmir_v23i11e31007_app3.docx]
