# Supplementary material for: Impact of Virtual Reality-Based Therapies on Cognition and Mental Health of Stroke Patients: Systematic Review and Meta-analysis
Source: J Med Internet Res. 2021 Nov 17;23(11):e31007. doi: 10.2196/31007 (PMC8663637; doi:10.2196/31007)
Supplement: Multimedia Appendix 4 [file jmir_v23i11e31007_app4.docx]

Egger’s funnel plot of publication bias.
